# The Association between Perpetuation of Intimate Partner Violence and Family Support on Couples with an Incarcerated Partner

**DOI:** 10.3390/ijerph191912165

**Published:** 2022-09-26

**Authors:** Eman Tadros, Selena D. Tate

**Affiliations:** 1 Division of Psychology and Counseling, Governors State University, University Park, IL 60441, USA; 2 Department of Agriculture, Nutrition, and Human Ecology, Prairie View A&M University, Prairie View, TX 77446, USA

**Keywords:** domestic violence, family support, incarceration, family

## Abstract

The ripple effect of incarceration is multifaceted. Studies show that imprisonment impacts the well-being of intimate and extended familial relationships, parental involvement, and financial support. Using data from the Multi-site Family Study on Incarceration, Parenting, and Partnering [MFS-IP], this study examined the effects of incarceration on families. The focus of this study is to examine the effect of family support on perpetrating physical violence. The findings revealed that the actor effect for men (*p* < 0.05) is statistically significant, meaning that men with less family support perpetuate higher levels of intimate partner violence (IPV). The partner effects for men to women (*p* < 0.05) is also statistically significant, meaning women with less family support coincide with men’s higher levels of IPV perpetration. Further, the effect of romantic attachment on perpetrating physical violence for men (*p* = 0.034) is statistically significant, meaning higher levels of romantic attachment towards the female partner coincides with their lower levels of perpetration of IPV. Clinical implications and future directions are provided.

## 1. Introduction

In 1930, the Federal Bureau of Prisons was established, and at its core was to act as a responsible custodian of incarcerated individuals. Then, the agency was responsible for 15 institutions, housing over 13,000 individuals [1]. While the focus is generally on the diversity disparity within the statistics surrounding incarceration rates, trends, and demographics, the social and emotional investments of families and communities play a critical role in the incarceration story. In addition to incarceration creating a disconnect between individuals and their communities, it impacts the family structure [2,3]. Several researchers have highlighted the role of families and the impact on these relationships [4,5,6,7,8,9,10,11]. The incarceration of individuals within the United States (U.S.) has a wide-ranging impact on the nation and its citizens. Therefore, examining trends in population, spending, and the effects on relationships, individuals, and families is key to better understanding the role of incarceration in the lives of Americans.

### 1.1. Family Support While Incarcerated

The social impact of incarceration (i.e., family relationships and support, health disparities, and mental health programs) serve as another component of the incarceration equation [5]. McKay, Lindquist et al. [2] examined the families of incarcerated individuals along a continuum, encompassing pre-incarceration, incarceration, and post-release. Amongst several findings, the authors noted complex relationships before incarceration. In numerous cases, there was minimal conflict, a host of positive experiences, and adequate financial support. However, several participants noted intimate partner violence and multiple arrests before incarceration. Additionally, Turney [11] identified the negative impact of incarceration on the quality of romantic relationships.

Adults who experience parental incarceration are another nuanced population in the incarceration narrative. The role of family support was highlighted by Luther [12], as the author specifically examined the topic of educational resilience amongst college-enrolled, adult children with incarcerated parents. The qualitative interviews revealed the importance of social support from attentive adults. Overall, these relationships supported resilience by promoting stability, pursuing education, and encouraging participants to live a different lifestyle than their parents. Various types of support were offered, including emotional support, goal envisioning, access to childhood activities (i.e., boys’ and girls’ clubs, athletics, and camps), and basic needs for housing, food, and clothing.

### 1.2. Challenges

Families employ significant financial, social, and emotional investments to sustain personal relationships with incarcerated individuals [4,5]. However, various challenges exist, including the financial drain of supporting incarcerated individuals [5,13] and the loss of financial support of the family due to incarceration [5,6,13,14]. For example, Geller et al. [6] examined the economic impact on children of incarcerated parents, focusing on the loss of financial support when fathers were in prison. Authors found that incarcerated men contributed $1300 less than men who had never been incarcerated. Additionally, there is a need for emotional and mental health assistance-support groups, expanded counseling services for families and the incarcerated, and therapy to address intimate relationship trust [4].

Maintaining consistent and complete communication posed a challenge to the incarcerated-family dynamic, as communication often decreases or is purposefully limited to reduce stress [5]. Exercising effective parenting also challenges the relationship between families and incarcerated individuals [3,15]. Tadros et al. [15] examined coparenting and mental health in families with an incarcerated parent. The authors noted that when caregivers reported a more significant number of externalizing symptoms, incarcerated parents perceived a less than ideal coparenting relationship. Feelings and behaviors impact the quality of the coparenting relationship, as the caregivers’ willingness and ability to participate are affected.

Incarcerated individuals do not complete their sentence alone, as the family experiences it with them [6,14]. Thus, the importance of maintaining positive family connections cannot be overstated. Often there is considerable work on behalf of the incarcerated men and partners to maintain contact via visits, phone calls, or letters [16]. In addition, there is significant financial strain due to the exorbitant cost of phone calls and visits [14] and the loss of income to support the family [6]. Additionally, illiteracy can hinder letter writing, and families experience stress associated with visits and procedures [16]. Lastly, there is a considerable strain on time and finances due to the distance between the facility and the family’s home [14,16,17].

### 1.3. Perpetuation of Violence

Intimate partner violence (IPV) describes but is not limited to physical aggression, sexual aggression, coercive behaviors [18], stalking, psychological abuse [19], emotional and financial abuse [20,21]. Recent statistics showed a 42% increase in IPV incidents and estimated that at least 10 million adults in the U.S. had experienced some form of IPV [22]. Statistics show that IPV impacts an individual’s mental and physical well-being [22], imposes negative economic problems, and is a catalyst for adverse outcomes in families [23].

Existing research has explored risk markers indicative of IPV perpetration and victimization [20,24,25,26]. Aggressive behavior against a former or current spouse/partner is a prevalent risk marker for male perpetration [20]. Such violent episodes are not random acts but account for aggression experienced during the onset and duration of the relationship [25,27]. At the same time, there are IPV-related risk markers distinctive to men and women [26]. For example, a recent study conducted a meta-analysis using a compilation of 503 studies that examined risk markers for IPV-related perpetration for men and women. The data highlighted specific risk markers for men, women, and both men and women. Of the factors discovered, several were categorized as the most robust risk markers. For women, the results revealed a history of causing injury to other individuals, prior physical perpetuation, sexual and emotional perpetuation, and physical, emotional, and sexual IPV victimization. The results also concluded that men had a history of inflicting injury to others and prior physical, emotional, sexual, stalking, and physical IPV perpetration.

Lastly, both men and women were likely to engage in alcohol or substance use, experience some form of childhood abuse, have indirect childhood exposure to IPV, be physically aggressive toward their offspring, and experience relationship issues [26]. Dixon and Graham-Kevan [20] and Lessard et al. [27] found similar risk markers conducive to the perpetration of IPV (i.e., aggressive tendencies toward non-family members, mental health problems, and substance abuse), which is consistent with other research. IPV violence is one of several risk factors that causes conflict in romantic and familial relationships.

### 1.4. Incarceration, Substance Use and Violence

Another area of concern is substance abuse. Previous literature asserts a correlation between IPV, substance abuse, and incarceration [24,27]. Using data from the National Violent Death Reporting System, Rizo et al. [24] conducted a study that investigated IPV-related homicides that were committed against individuals released from imprisonment. Attention was given to understanding victim risk markers and other challenges. The victims’ background revealed previous criminal activity and engagement in IPV-related incidents. The findings also showed that verbal disputes, physical altercations, alcohol consumption, and drug use were issues that precipitated and influenced IPV-related homicidal incidents. Saxena and Messina [28] echoed similar findings in their study that explored the experiences of incarcerated women. Of the participants, it was found that a significant number of the women had committed violent offenses, theft, and reported use of at least two or more substances within a year before imprisonment. Criminal activity during adolescence, childhood victimization, and substance use predicted female perpetuation of IPV and aggression toward other individuals. Shorey et al. [29] next explored men with a criminal history of IPV. The subjects were referred to and mandated to participate in domestic violence prevention programs. Several measurements were used to ascertain the correlation between marijuana use, IPV perpetuation, alcohol use and problems, and antisocial personality symptoms. Overall, the results concluded that the use of marijuana is predictive of three types of IPV: psychological, physical, and sexual violence. The results of the previous studies support literature that asserts substance use is a risk marker closely related to the perpetuation of violence and incarceration.

### 1.5. The Current Study

The focus of this study is the investigation of the effect of family support on the perpetration of physical violence. We used the Actor-Partner Interdependence Modeling (APIM) Cook and Kenny [30] to evaluate the impact of the independent variable, family support, as well as the covariates (romantic attachment and childhood stability), on the partner’s dependent variable on perpetrating physical violence (partner effects). The following questions are examined:

1. Do individuals with less family support perpetuate more physical violence?
2. Is having a partner with less family support associated with perpetuating more physical violence?

Corresponding with the research questions, we have two hypotheses. First, we hypothesize a negative relationship between the variables, with less family support being associated with higher levels of perpetuating violence for actors, when controlling for the potential confounding effects of romantic attachment and childhood stability. Second, we hypothesize a negative relationship between family support and perpetuating violence, with those whose partners perceive themselves to have more family support to engage in the perpetuation of physical violence when controlling for the potential confounding effects of romantic attachment and childhood stability.

## 2. Materials and Methods

### 2.1. Data Source

Examining incarcerated men and their female coparents from Indiana, Ohio, Minnesota, New Jersey, and New York, this study sought to understand the dynamics of maintaining family relationships. Using data from the Multi-site Family Study on Incarceration, Parenting, and Partnering [MFS-IP] [31], partners were interviewed between December 2008 and August 2014. Several participant conditions existed, including age requirements and fluency in the English language. Various recent studies have utilized this dataset to examine family dynamics [2,4,7,8,9,32,33,34,35].

### 2.2. Measures

#### 2.2.1. Family Support

Our independent variable of interest was family support. Family support was measured by six five-point Likert scale questions surrounding their perception of their relationship with family members. These family members were outside of their identified partner and focal child. Questions consisted of rating agreement level (strongly agree to strongly disagree) regarding feeling close to family, family members standing by you, family involved in your life, and being criticized by family. The family support scale has a high internal consistency or reliability. It has a Cronbach’s alpha of 0.866 for the non- incarcerated women and 0.871 for the incarcerated men. Further, a recent study that analyzed family support with female coparenting partners of incarcerated men used this scale [7].

#### 2.2.2. Perpetrating Physical Violence

Our dependent variable of interest was perpetrating physical violence. The question surrounding perpetrating physical violence asked, “During the 6 months before you were incarcerated, how often did you physically hurt or get rough with a spouse or partner or other family members when you had been drinking or using drugs?” The answer options were often, sometimes, rarely, or never.

#### 2.2.3. Covariate: Childhood stability

The first covariate, childhood stability, was made up of the question, “Overall, how stable do you feel your parenting arrangement was during your childhood?” Answer options were very stable, stable, unstable, and very unstable.

#### 2.2.4. Covariate: Romantic Attachment

The second covariate, romantic attachment, consisted of four items rated by participants on how much they agree or disagree with the statements in reference to close relationships. See Table 1.

#### 2.2.5. Data Analysis

In this study, we utilized Structural Equation Modeling (SEM) with maximum likelihood estimation (APIM_SEM). APIM is the best methodological analytic tool for examining two-person relationships because it is designed to measure interdependence within interpersonal relationships [30]. We used APIM to evaluate the impact of family support and the covariates (romantic attachment and childhood stability) on their partner’s perpetuation of physical violence.

## 3. Results

The total number of dyads was *n =* 866. The number of cases for men on family support *n =* 866, and for perpetrating physical violence, *n =* 704. For women, the number of cases on family support is *n =* 865, and on perpetrating physical violence, *n =* 698. 

### 3.1. Actor Effects

**Figure 1:**
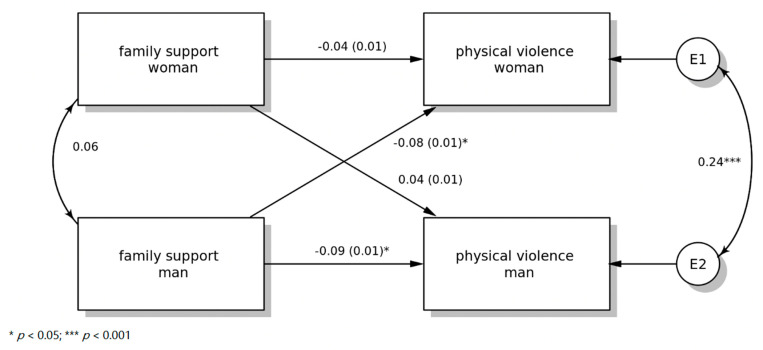
Model with standardized parameter estimates.

The total number of dyads is 866. The overall actor effect is statistically significant (*p* < 0.05) equaling −0.009. The actor effect for the men is statistically significant, equaling −0.012, with the overall standardized effect equaling −0.086. The actor effect for the women is not statistically significant (*p* = 0.329) equaling −0.006 with an overall standardized actor effect of −0.040. See Figure 1. 

### 3.2. Partner Effects

The overall partner effect is not statistically significant (*p* = 0.425), equal to −0.003. The partner effect from men to women is statistically significant (*p* < 0.05), equal to −0.011 with an overall standardized effect of −0.076. The partner effect from women to men is not statistically significant (*p* = 0.355), equal to 0.005 with an overall standardized effect of 0.034. See Figure 1. 

### 3.3. Childhood Stability

The effect of childhood stability on perpetrating physical violence for men is not statistically significant (*p* = 0.605), equal to −0.012 with an overall standardized effect of −0.02. For women, the effect of childhood stability on perpetrating physical violence is not statistically significant (*p* = 0.980), with an overall standardized effect of −0.001. See Figure 1. 

### 3.4. Romantic Attachment

The effect of romantic attachment on perpetrating physical violence for men is statistically significant (*p* = 0.034) and equal to −0.012, with an overall standardized effect of −0.078. For women, the effect of romantic attachment on perpetrating physical violence is not statistically significant (*p* = 0.933), with an overall standardized effect of 0.003. See Figure 1. 

## 4. Discussion

Previous research acknowledges that families encounter challenges when a family member is incarcerated [11,23,36,37]. Managing a romantic relationship or delivering parental support can become problematic for an incarcerated individual and the non-incarcerated partner. The focus of this study is to examine the effect of family support on perpetrating physical violence. Research shows that men and women perpetuate physical aggression [20,26]. However, it is essential to note that men and women have similar and differing risk markers [26]. In the literature, family connectedness is a significant source of support for incarcerated individuals [16,38]. The factors of familial support vary, as do the needs of the individual [39]. According to the first finding, our results indicated that actor effects for men are statistically significant. Explicitly, it confirms the connection between family support and the perpetuation of IPV. Moreover, this study’s results concluded that men with low levels of family involvement perpetuate higher levels of IPV. Although it is unclear what support factors were demonstrated, the findings of prior studies confirm that familial support has a positive outcome when dealing with criminal recidivism [40] and reincarceration [39].

Our second finding revealed that the partner effect for men to women is statistically significant. Specifically, higher levels of familial support are more likely to decrease the occurrence of IPV. In contrast, the partner effect for women to men was not significant. The finding is inconsistent with past studies investigating the occurrence and risk makers associated with intimate partner violence. Instead, results from these studies claimed that family support is a protective marker that reduces the perpetuation of domestic violence. For example, one study found that mothers with young children with higher levels of family support were less likely to experience intimate partner violence [41]. In another study, researchers found that social support from family (e.g., intimate partners), friends, other inmates, and professionals were a source of emotional support for incarcerated women [42]. Perhaps the contrast between the results of this study and previous works is the claim that some women with severe histories of violence perpetuate violence toward male partners as self-defense or a never-again stance [43].

Multiple studies have identified and examined robust risk markers predictive of intimate partner violence [20,24,25,26,29,44]. These findings provide crucial information for understanding and preventing the occurrence of IPV [44]. Using a meta-analysis, Keilholtz et al. [44] examined several life stressors as risk markers for IPV. The results found that relational distress strongly predicted IPV and victimization. Similarly, the third finding of this study suggests that higher levels of romantic attachment [relationship satisfaction] decrease the perpetration of IPV for men toward their female partners [36]. The literature has also confirmed that relationship satisfaction is a protective risk marker for men to women and women to men [45,46,47]; surprisingly, our results for women and their male partners were unsupportive. Duration of the relationship, severity of the violence, and gender differences are plausible explanations for the opposing result; however, more research in this area is compulsory [45,47].

Previous research asserts that childhood exposure to IPV is a standard risk marker for several aversive outcomes. More importantly, it postulates that childhood exposure to IPV predicts IPV perpetration in adulthood [48,49,50,51]. Interestingly, our fourth finding was not statistically significant. The finding suggests that childhood stability is not predictive of adult IPV perpetration. Although it is unclear why our results are conflicting, it may be plausible to assume that other factors may explain the inconsistencies. Saxena and Messina [28] confirmed that various types of abuse (i.e., child abuse) contributed to adult IPV and victimization. However, the researchers also allege that early criminal involvement before age 18 was the strongest predictor of adult IPV. Similarly, Widom et al. [52] findings differed from existing literature. Their surprising results suggest that childhood experiences of abuse are not associated with adult IPV when examining gender differences independently. Such findings warrant further research that explores gender differences and the perpetuation of IPV.

### 4.1. Clinical Implications

It is imperative for marriage and family therapists (MFTs) to understand the challenges associated with incarceration, relationship quality, and family support. Asking the right questions will help MFTs assess relational patterns, levels of stress/anxiety, and track the history of violence preceding incarceration. Likewise, understanding and identifying risk markers of IPV perpetration and victimization will encourage MFTs to be observant [25], support coping skills and identify unhealthy relational dynamics. An assessment tool that may prove helpful for gathering information about coping skills, family functioning, support systems, and tracking patterns and themes is a basic genogram [53,54]. There continues to be a call for more programs and policies to support families while their loved ones are incarcerated. Suggested interventions include additional parenting classes, support groups for women with incarcerated partners, and funding to alleviate the burden of phone calls and visits. Other suggestions include housing individuals close to families, alleviating stressors surrounding the visit, support and education for caregivers, and generally more programs to support and strengthen the family ties during incarceration [2,10,12,15,17,32,55,56].

### 4.2. Limitations

Researchers acknowledge several limitations of the current study, including utilizing secondary data from the Multi-site Family Study on Incarceration, Parenting, and Partnering [MFS-IP] [31] and its limiting dataset. The MFS-IP fails to support generalization and does not represent the national population. Additionally, the dataset did not include same-sex couples and incarcerated mothers who have a nonincarcerated male partner. The data from the MFS-IP only addressed the relationships between incarcerated men and nonincarcerated women. The data collection occurred from December 2008 through April 2014, thus, the data may be considered a bit dated. There are likely newer developments, challenges, and issues prevalent in this population that the data does not reflect. For example, the omission of the effects of COVID-19 on the incarcerated population as it was not present during this time frame. The severity of the coronavirus pandemic forced correction centers to suspend in-person visitations to control the spread of the virus [56,57]. Therefore, one can assume this disruption interfered with families staying connected and supported. Finally, a measurement tool was used to assess the perpetration of physical violence; however, it is unclear how often the violence occurred.

### 4.3. Future Directions

The topic of incarceration within the U.S. is multifaceted, as it impacts not only the incarcerated individuals but also their families and romantic relationships. Intimate partner violence is a known risk marker that jeopardizes the well-being of intimate relationships [25,27]. Given our quantitative results, future studies should advance the field by conducting qualitative studies exploring the family of origin to investigate patterns and behaviors that could impede intimate relationships and familial dynamics. A systemic approach to consider is the Bowen Family Systems Theory [53,54]. The model explores types of anxiety exhibited in families, and how it influences the family’s emotional functioning. Bowen’s theoretical model also focuses on the multigenerational transmission of symptoms and behaviors (i.e., family violence) transmitted from generation to generation. Focusing on behaviors and patterns of the family of origin may give insight into how incarcerated individuals and their families manage stressful situations and assist with identifying viable support systems and interventions.

Furthermore, it may help preclude the occurrence or the recidivism of intimate partner violence in intimate relationships. Future research exploring same-sex couples, incarcerated mothers, and their nonincarcerated partners is also needed to expand the literature on incarcerated populations. Finally, research exploring the impact of COVID-19 is needed to understand the relational effects of suspended in-person visitations, phone privileges, and other means of contact with intimate partners and other family members [58].

## 5. Conclusions

Imprisonment affects incarcerated individuals and their families. The findings from this study suggest that the actor effect for men is statistically significant: men with less family support perpetuate higher levels of IPV, and women with less family support correspond with higher levels of IPV perpetration for men. Conversely, the partner effects for women to men were not significant. Although the literature supports most of the research findings, future research is still needed to understand the gender differences concerning the effects of intimate partner violence, familial support, and relationship satisfaction with incarcerated populations.

## Figures and Tables

**Table 1:** Romantic Attachment Questions.

1.	It is easy for me to become emotionally close to others. I am comfortable depending on them and having them depend on me. I don’t worry about being alone or having others not accept me.
2.	I am uncomfortable getting close to others. I want emotionally close relationships, but I find it difficult to trust others completely, or to depend on them. I worry that I will be hurt if I allow myself to become too close to others.
3.	I want to be completely emotionally intimate with others, but I often find that others are reluctant to get as close as I would like. I am uncomfortable being without close relationships, but I sometimes worry that others don’t value me as much as I value them.
4.	I am comfortable without close emotional relationships. It is very important to me to feel independent and self-sufficient, and I prefer not to depend on others or have others depend on me.

Scale-Strongly agree, Agree, Disagree, or Strongly disagree.

## Data Availability

The data is publicly available on ICPSR.
